# Hospitalizations, emergency medical care utilization, and contacts with the regional on-call medical services among nursing home residents in Germany: a cross-sectional study in 44 nursing homes

**DOI:** 10.1186/s12913-025-12342-3

**Published:** 2025-02-01

**Authors:** Paula Lienesch, Heinz Rothgang, Ansgar Gerhardus, Karin Wolf-Ostermann, Falk Hoffmann, Jonas Czwikla

**Affiliations:** 1 Department of Health, Long-Term Care and Pensions, SOCIUM Research Center On Inequality and Social Policy, University of Bremen, Mary-Somerville-Straße 5, Bremen, 28359 Germany; 2 High-Profile Area of Health Sciences, University of Bremen, Bibliothekstraße 1, Bremen, 28359 Germany; 3 Department for Health Services Research, Institute of Public Health and Nursing Research (IPP), University of Bremen, Grazer Straße 4, Bremen, 28359 Germany; 4 Department for Health Care Research, Institute of Public Health and Nursing Research (IPP), University of Bremen, Grazer Straße 4, Bremen, 28359 Germany; 5 Department of Health Services Research, School of Medicine and Health Sciences, Carl von Ossietzky Universität Oldenburg, Ammerländer Heerstraße 114-118, Oldenburg, 26129 Germany

**Keywords:** Acute medical care, Hospitalization, Emergency medical care, On-call medical service, Long-term care, Nursing home, Nursing home resident, Health services research

## Abstract

**Background:**

Nursing home residents frequently utilize medical care, but there lacks a complete picture of their acute medical care utilization. We quantified hospitalizations, emergency medical care utilization, and contacts with the regional on-call medical services among nursing home residents, and investigated individual characteristics that may be associated with the utilization of these medical care types.

**Methods:**

Cross-sectional data from the “Needs-based provision of medical care to nursing home residents” (MVP-STAT) study were analyzed, which were collected in 44 German nursing homes from 442 residents in 2018/2019. Proportions of residents with at least one hospitalization, emergency medical care utilization (via the nationwide phone number 112), and contact with an on-call medical service (nationwide via 116117) over the previous 12 months were determined. Associations between individual characteristics and the utilization of the three medical care types were examined using multivariable logistic regressions.

**Results:**

Of the analyzed residents, 45.8% were hospitalized, 23.2% utilized emergency medical care, and 12.1% had contact with an on-call medical service at least once in the previous 12 months. Hospitalizations were positively associated with male vs. female sex (adjusted odds ratio 1.99 [95% confidence interval 1.22–3.26]), age group 85 + vs. 60–74 years (2.15 [1.12–4.13]), long-term care grades 4/5 vs. 1/2 (2.78 [1.48–5.21]), 6 + vs. 0–1 Elixhauser diseases (2.58 [1.01–6.62]), and the risk or presence of vs. no malnutrition (3.10 [1.52–6.35] and 2.01 [1.26–3.21]); and not associated with years of residence in the respective nursing home. Emergency medical care utilization was positively associated with age group 85 + vs. 60–74 years (2.58 [1.14–5.84]) and long-term care grades 3 and 4/5 vs. 1/2 (2.65 [1.07–6.55], 6.31 [2.60–15.35]); negatively associated with 5 + vs. 1- < 3 years of residence (0.46 [0.24–0.86]); and not associated with sex, the number of Elixhauser diseases, and nutritional status. No associations were found with on-call medical services.

**Conclusions:**

Hospitalizations and emergency medical care utilization were more frequent among nursing home residents than contacts with on-call medical services. Future studies should investigate whether the frequent hospitalizations and emergency medical care utilization among nursing home residents are justified, or whether they can be reduced by strengthening medical care provision by on-call doctors and other professionals.

**Trial registration:**

DRKS00012383 [2017/12/06].

## Background

Caring for older people in need of long-term care (LTC) is an essential part of healthcare, especially in view of demographic change and an ageing population [1]. The proportion of individuals aged 65 years and older grew across all European Union member states from 16% in 2003 to 21% in 2023 [2]. Due to this rise, the number of LTC dependents is also rising [3]. In 2023, 5.58 million people in Germany met the requirements for LTC; of these 16% were nursing home residents (84% were community-dwelling LTC dependents) [4].

LTC dependents, and in particular nursing home residents, often have complex medical care needs arising from multimorbidity [5], frailty [6], cognitive impairments [7], and polypharmacy [8], which increase the risk of acute medical care utilization. Previous international [9–11] and German [12–14] studies showed that nursing home residents are more likely to be hospitalized and utilize emergency medical care than community-dwelling older adults. This might be justified by different medical care needs, but it also appears that some hospitalizations and emergency medical care utilization among nursing home residents might be avoidable [15–18], and this is especially the case in Germany [12, 19–22].

Individual characteristics, such as sex, age, and morbidity, that may be associated with hospitalizations and emergency medical care utilization, have already been investigated internationally and nationally [23–29]. Although previous German studies investigated contacts between nursing home residents and general practitioners and medical specialists [30–34], contacts with regional on-call medical services [35] have not yet been sufficiently explored. In Germany, on-call medical services provide care in non-life-threatening situations outside standard consulting hours. They are available around the clock via the nationwide phone number 116117 and provide acute medical care either in on-call practices or – if necessary – the patient’s home [35]. By contrast, emergency medical care is explicitly intended for life-threatening situations. It is requestable nationwide, around the clock, via the European emergency number 112 [36] and may result in ambulance dispatch and transport to an emergency department. Both on-call practices and emergency departments can also be visited without calling 116,117 and 112, respectively.

Another research gap relates to factors that potentially influence hospitalizations, emergency medical care utilization, and contacts with on-call medical services. These factors have not yet been systematically compared between the three medical care types, but such a comparison is crucial to gain a full overview of acute medical care utilization among nursing home residents. By better understanding the interrelationships between residents’ individual characteristics and utilization patterns it may be possible to develop interventions to reduce avoidable hospitalizations and emergency medical care utilization.

The aims of this research were i) to quantify hospitalizations, emergency medical care utilization, and contacts with the regional on-call medical services among nursing home residents in Germany, and ii) to examine whether hospitalizations, emergency medical care utilizations and contacts with the on-call medical services are associated with individual characteristics of residents.

## Methods

### Design and setting

This research used cross-sectionally collected data from the German mixed-methods study “Needs-based provision of medical care to nursing home residents” (MVP-STAT), which is described in detail in a study protocol [37] and other MVP-STAT publications [30, 31, 38, 39]. In brief, the overall aim of MVP-STAT was to gain insights into the appropriateness of medical care for nursing home residents and to propose solutions to improve medical care where it is inappropriate. The cross-sectional data were collected from residents of nursing homes in the Free Hanseatic City of Bremen and the surrounding area of the federal state of Lower Saxony between February 2018 and March 2019. To reach the target sample size of 500 residents [37], all nursing homes in Bremen (*n* = 87) and a convenience sample of homes in Lower Saxony (*n* = 262) were invited, of which 44 (12.6% response) participated. These facilities invited all eligible residents (about 2124 LTC dependents, who were aged 60 + and had resided in the respective nursing home for at least 12 months) or their relatives/legal guardians to take part in MVP-STAT; 442 (20.8% response) provided informed consent and participated. The standardized data collection was performed by trained study nurses who, inter alia, reviewed nursing records and interviewed residents’ care nurses [37].

A positive ethics vote for MVP-STAT was granted by the Ethics Committee of the University of Bremen on 23 November 2017 (Ethics Committee Number “MVP-STAT”).

### Variables

Data on hospitalizations (irrespective whether planned [elective] or not and regardless whether residents were directly admitted from nursing homes or via emergency departments) in the previous 12 months were collected from the nursing records. Data on emergency medical care utilization (112; irrespective whether it took place in nursing homes/ambulances and/or emergency departments) were also collected from the nursing records of the previous 12 months. The same applied for data on contacts with the regional on-call medical services (116117; regardless whether they took place in nursing homes and/or on-call practices). Besides dichotomous information on whether the respective medical care type had been utilized in the previous 12 months, the collected data included the start and end dates of hospitalizations as well as dates of emergency medical care utilization and contacts with the on-call medical services.

Information on sex, age, LTC grade, years of residence in the respective nursing home, and diagnoses were also collected from the nursing records. In Germany, LTC grades are assessed by the Medical Advisory Service. They are segmented into five distinct grades: 1 = low limitations, 2 = substantial limitations, 3 = severe limitations, 4 = very severe limitations, and 5 = very severe limitations with special nursing care requirements [40]. The diagnoses were available in text form and in most cases also coded according to the International Classification of Diseases, 10th Revision (ICD-10), German Modification [41]. Where ICD-10 codes were not available, they were subsequently coded by qualified project staff. The diagnoses were used to obtain information on the 31 diseases included in the Elixhauser comorbidity measure, which is widely used in health services research [42, 43].

In addition, the nutritional status of residents was proxy-assessed by residents’ care nurses using the established Mini Nutritional Assessment Short-Form (MNA-SF) tool. The MNA-SF uses six questions on appetite loss, weight loss, mobility, stress/acute disease, dementia/depression, and body mass index or calf circumference to differentiate between normal nutritional status (12–14 MNA-SF points), risk of malnutrition (8–11 MNA-SF points), and malnutrition (0–7 MNA-SF points) [44–47].

### Statistical analysis

First, the distributions of the individual characteristics sex (female, male), age (60–74, 75–84, and 85 + years), LTC grade (1/2 [less severe limitations], 3 [severe limitations], and 4/5 [very severe limitations]), years of residence (1- < 3, 3- < 5, and 5 + years), number of Elixhauser diseases (0–1, 2–3, 4–5, and 6 +), and nutritional status (normal, risk of malnutrition, malnutrition) were investigated. For age and the number of Elixhauser diseases, mean values and standard deviations (SDs) were also examined.

Second, proportions of residents with at least one hospitalization, emergency medical care utilization, and contact with an on-call medical service within the previous 12 months were calculated along with corresponding 95% confidence intervals (CIs). Furthermore, the mean numbers and SDs of hospitalizations, emergency contacts, and contacts with the on-call medical services among residents with at least one utilization of the respective medical care type were determined. Regarding hospitalizations, these values were also determined for the duration of hospital stay. All calculations were also performed in relation to the individual characteristics of the residents.

Third, three multivariable logistic regressions were performed to examine whether the utilization of the three medical care types is associated with individual characteristics of residents. In these regressions, hospitalizations, emergency medical care utilization, and contacts with the on-call medical services served as dichotomous dependent variables (coded as yes or no). The independent variables were all individual characteristics examined in steps one and two. In each model, odds ratios (ORs) along with corresponding 95% CIs and p-values were calculated to identify statistically significant associations.

All analyses were performed using SAS 9.4 (SAS Institute Inc., Cary, NC, USA) [48].

## Results

### Characteristics of nursing home residents

The analyzed study population comprised 406 residents of 44 nursing homes which were heterogenous in terms of location (58% from the urban area of Bremen and 42% from the predominantly rural surrounding area of Lower Saxony), ownership (51% independent nonprofit, 44% private, 5% public/municipal), and size (mean number of nursing home beds 87.2 [SD 48.8]). 36 individuals were excluded from the initial population of 442 due to invalid information on age (*n* = 8) or duration of residence (*n* = 28). The distribution by sex shows that 66.5% of the residents were female (Table 1). The mean age was 83.1 (SD 9.4) years, 42.5% residents had an LTC grade of four or five, and 50.3% had resided in the respective nursing home for less than three years. The mean number of Elixhauser Diseases was 2.6 (SD 1.7), and almost half of the residents were malnourished (12.3%) or at risk of malnutrition (36.0%).

**Table 1:** Characteristics of the study population

Characteristic	N	%
Sex (*n* = 406)		
Female	270	66.5
Male	136	33.5
Age group (*n* = 406)		
60–74 years	76	18.7
75–84 years	136	33.5
85 + years	194	47.8
Long-term care grade (*n* = 400)		
1/2^a^	79	19.8
3	151	37.8
4/5^b^	170	42.5
Years of residence (*n* = 406)		
1- < 3 years	204	50.3
3- < 5 years	89	21.9
5 + years	113	27.8
Number of Elixhauser Diseases (*n* = 406)		
0–1	104	25.6
2–3	190	46.8
4–5	86	21.2
6 +	26	6.4
Mini Nutritional Assessment (*n* = 397)		
Normal nutritional status	205	51.6
Risk of malnutrition	143	36.0
Malnutrition	49	12.3

^a^Two residents had long-term care grade 1 and 77 had grade 2

^b^121 residents had long-term care grade 4 and 49 had grade 5

### Hospitalizations

Overall, 45.8% (*n* = 186) of the 406 residents had been hospitalized at least once in the previous 12 months (Table 2). The proportions of hospitalized residents among men and older individuals were higher than among women and younger individuals, but the differences were not statistically significant. The proportion of hospitalized individuals increased significantly from 30.4% for residents with LTC grades 1/2 to 57.1% for those with LTC grades 4/5. Hospitalizations tended to decrease with increasing years of residence in the respective nursing home, and to increase with an increasing number of Elixhauser diseases, although these trends were not significant. Malnourished residents and those at risk of malnutrition had higher proportions of hospitalized individuals than those with normal nutritional status (63.3% and 54.6% vs. 34.2%).

**Table 2:** Hospitalizations among nursing home residents in the previous 12 months

Characteristic		At least one hospitalization		Number of hospitalizations among those with at least one hospitalization	Length of hospitalizations in days among those with at least one hospitalization
	N	% (95% CI)	N	Mean (SD)	Mean (SD)
Total (*n* = 406)	406	45.8 (41.0–50.7)	175	1.6 (1.0)	6.0 (9.8)
Sex (*n* = 406)					
Female	270	43.0 (37.2–48.9)	109	1.6 (1.0)	5.7 (8.9)
Male	136	51.5 (43.2–59.7)	66	1.7 (0.9)	6.6 (11.0)
Age group (*n* = 406)					
60–74 years	76	38.2 (28.1–49.4)	28	1.6 (1.3)	8.6 (11.3)
75–84 years	136	48.5 (40.3–56.9)	61	1.8 (1.0)	5.9 (10.6)
85 + years	194	46.9 (40.0–53.9)	86	1.5 (0.9)	5.3 (8.2)
Long-term care grade (*n* = 400)					
1/2	79	30.4 (21.3–41.2)	21	1.5 (0.6)	7.1 (10.1)
3	151	43.1 (35.4–51.0)	63	1.5 (0.8)	8.3 (14.0)
4/5	170	57.1 (49.5–64.3)	91	1.7 (1.1)	4.5 (5.5)
Years of residence (*n* = 406)					
1- < 3 years	204	48.5 (41.8–55.4)	96	1.7 (0.9)	5.5 (7.3)
3- < 5 years	89	44.9 (35.0–55.3)	35	1.7 (1.3)	5.8 (9.0)
5 + years	113	41.6 (32.9–50.8)	44	1.5 (0.8)	7.7 (14.5)
Number of Elixhauser Diseases (*n* = 406)					
0–1	104	38.5 (29.7–48.1)	37	1.4 (0.9)	5.0 (9.4)
2–3	190	47.9 (40.9–55.0)	85	1.6 (0.9)	6.2 (10.2)
4–5	86	46.5 (36.4–57.0)	38	1.8 (1.3)	5.4 (7.6)
6 +	26	57.7 (39.0–74.5)	15	1.7 (0.9)	9.3 (12.9)
Mini Nutritional Assessment (*n* = 397)					
Normal nutritional status	205	34.2 (28.0–40.9)	66	1.4 (0.8)	5.8 (9.4)
Risk of malnutrition	143	54.6 (46.4–62.5)	74	1.7 (1.1)	6.4 (11.4)
Malnutrition	49	63.3 (49.3–75.3)	28	1.9 (1.1)	5.9 (6.5)

*Abbreviations: CI* confidence interval, *SD* standard deviation

Residents with at least one hospitalization had a mean number of 1.6 hospitalizations in the previous 12 months, differing only slightly by individual characteristics. The mean length of hospitalization was 6.0 days. It was comparatively high in the youngest age group, among residents with LTC grades 1/2 or 3, among those who had already spent 5 + years in the respective nursing home, and among individuals with 6 + Elixhauser diseases.

### Emergency medical care utilization

Overall, 23.2% (*n* = 94) of 405 residents (for one resident, information on emergency medical care utilization was missing) had utilized at least one emergency medical care in the previous 12 months (Table 3). The proportions of residents with emergency medical care utilization differed only slightly by sex, but increased by age, although not statistically significant. A significant increase in the proportion with utilization was observed from 10.1% for individuals with LTC grades 1/2 to 34.1% for those with LTC grades 4/5. Utilization tended to decrease with increasing years of residence in the respective nursing home, differed only slightly by the number of Elixhauser diseases, and tended to be comparatively high among residents with malnutrition.

**Table 3:** Emergency medical care utilization among nursing home residents in the previous 12 months

Characteristic		At least one emergency medical care utilization		Number of emergency contacts among those with at least one emergency medical care utilization
	N	% (95% CI)	N	Mean (SD)
Total (*n* = 405)	405	23.2 (19.4–27.6)	94	1.3 (0.7)
Sex (*n* = 405)				
Female	270	22.6 (18.0–28.0)	61	1.2 (0.7)
Male	135	24.4 (18.0–32.3)	33	1.5 (0.7)
Age group (*n* = 405)				
60–74 years	75	14.7 (8.4–24.4)	11	1.8 (1.1)
75–84 years	136	25.0 (18.5–32.9)	34	1.3 (0.5)
85 + years	194	25.3 (19.7–31.8)	49	1.2 (0.7)
Long-term care grade (*n* = 399)				
1/2	79	10.1 (5.2–18.7)	8	1.4 (0.7)
3	150	18.7 (13.2–25.7)	28	1.2 (0.4)
4/5	170	34.1 (27.4–41.5)	58	1.4 (0.8)
Years of residence (*n* = 405)				
1- < 3 years	204	27.5 (21.8–34.0)	56	1.3 (0.6)
3- < 5 years	89	22.5 (15.0–32.2)	20	1.5 (1.1)
5 + years	112	16.1 (10.4–24.0)	18	1.2 (0.4)
Number of Elixhauser Diseases (*n* = 405)				
0–1	104	24.0 (16.9–33.1)	25	1.4 (0.9)
2–3	189	23.3 (17.8–29.8)	44	1.3 (0.6)
4–5	86	20.9 (13.7–30.7)	18	1.3 (0.8)
6 +	26	26.9 (13.7–46.1)	7	1.1 (0.4)
Mini Nutritional Assessment (*n* = 396)				
Normal nutritional status	204	18.1 (13.5–24-0)	37	1.2 (0.5)
Risk of malnutrition	143	27.3 (20.6–35.1)	39	1.4 (0.7)
Malnutrition	49	32.7 (21.2–46.6)	16	1.5 (1.1)

*Abbreviations: CI* confidence interval, *SD* standard deviation

The mean number of emergency contacts among residents with at least one emergency medical care utilization was 1.3 and differed only slightly by individual characteristics.

### Contacts with regional on-call medical services

Overall, 12.1% (*n* = 49) of 406 residents had had contact with an on-call medical service at least once in the previous 12 months (Table 4). This proportion differed only slightly by sex and age but tended to increase by LTC grade and decrease by years of residence, although not statistically significant. Differences in the proportion with contact were minimal in terms of the number of Elixhauser diseases and nutritional status.

**Table 4:** Contacts with regional on-call medical services among nursing home residents in the previous 12 months

Characteristic		At least one contact with an on-call medical service		Number of contacts with on-call medical services among those with at least one contact
	N	% (95% CI)	N	Mean (SD)
Total (*n* = 406)	406	12.1 (9.3–15.6)	49	1.2 (0.5)
Sex (*n* = 406)				
Female	270	11.5 (8.2–15-8)	31	1.3 (0.5)
Male	136	13.2 (8.5–20.0)	18	1.2 (0.4)
Age group (*n* = 406)				
60–74 years	76	10.5 (5.4–19.4)	8	1.0 (0.0)
75–84 years	136	13.2 (8.5–20.0)	18	1.4 (0.6)
85 + years	194	11.9 (8.0–17.2)	23	1.2 (0.4)
Long-term care grade (*n* = 400)				
1/2	79	8.9 (4.4–17.2)	7	1.0 (0.0)
3	151	10.6 (6.6–16.5)	16	1.4 (0.6)
4/5	170	15.3 (10.7–21.5)	26	1.2 (0.4)
Years of residence (*n* = 406)				
1- < 3 years	204	14.7 (10.5–20.2)	30	1.3 (0.5)
3- < 5 years	89	12.4 (7.0–20.8)	11	1.3 (0.5)
5 + years	113	7.1 (3.6–13.4)	8	1.1 (0.4)
Number of Elixhauser Diseases (*n* = 406)				
0–1	104	12.5 (7.5–20.2)	13	1.2 (0.4)
2–3	190	11.1 (7.3–16.3)	21	1.3 (0.6)
4–5	86	14.0 (8.2–22.8)	12	1.1 (0.3)
6 +	26	11.5 (4.0–29.0)	3	1.3 (0.6)
Mini Nutritional Assessment (*n* = 397)				
Normal nutritional status	205	10.2 (6.8–15.2)	21	1.1 (0.4)
Risk of malnutrition	143	14.7 (9.8–21.4)	21	1.2 (0.5)
Malnutrition	49	12.2 (5.7–24.2)	6	1.7 (0.5)

*Abbreviations: CI* confidence interval, *SD* standard deviation

The mean number of contacts with on-call medical services for residents with at least one contact was 1.2 and differed only slightly by individual characteristics.

### Multivariable analysis

Regarding hospitalizations, male vs. female residents were significantly more likely to be hospitalized (OR 1.99 [95% CI 1.22–3.26]) (Table 5). Furthermore, individuals aged 85 + vs. 60–74 years (2.15 [1.12–4.13]), those with LTC grades 4/5 vs. 1/2 (2.78 [1.48–5.21]), and those with 6 + vs. 0–1 Elixhauser diseases (2.58 [1.01–6.62]) had a higher chance of being hospitalized. Hospitalizations were also more likely among residents at risk of malnutrition and those with malnutrition vs. the reference group (3.10 [1.52–6.35], 2.01 [1.26–3.21]). No associations were found with years of residence in the respective nursing home.

**Table 5:** Multivariable logistic regressions

Characteristic	At least one hospitalization^a^		At least one emergency medical care utilization^b^		At least one contact with an on-call medical service^c^	
	OR (95% CI)	*p*-value	OR (95% CI)	*p*-value	OR (95% CI)	*p*-value
Sex						
Male (ref. female)	**1.99 (1.22–3.26)**	**0.0062**	1.55 (0.88–2.73)	0.1321	1.12 (0.56–2.24)	0.7409
Age group (ref. 60–74 years)						
75–84 years	1.73 (0.91–3.30)	0.0956	2.16 (0.96–4.88)	0.0642	1.20 (0.47–3.07)	0.7050
85 + years	**2.15 (1.12–4.13)**	**0.0218**	**2.58 (1.14–5.84)**	**0.0236**	1.09 (0.42–2.80)	0.8651
Long-term care grade (ref. 1/2)						
3	1.84 (0.99–3.43)	0.0553	**2.65 (1.07–6.55)**	**0.0346**	1.15 (0.44–3.02)	0.7788
4/5	**2.78 (1.48–5.21)**	**0.0014**	**6.31 (2.60–15.35)**	** < 0.0001**	1.89 (0.75–4.80)	0.1786
Years of residence (ref. 1- < 3 years)						
3- < 5 years	0.84 (0.49–1.45)	0.5250	0.76 (0.41–1.42)	0.3960	0.84 (0.39–1.79)	0.6530
5 + years	0.74 (0.44–1.23)	0.2419	**0.46 (0.24–0.86)**	**0.0157**	0.44 (0.19–1.01)	0.0519
Number of Elixhauser Diseases (ref. 0–1)						
2–3	1.62 (0.95–2.75)	0.0767	1.20 (0.65–2.22)	0.5617	0.88 (0.41–1.87)	0.7303
4–5	1.46 (0.78–2.74)	0.2425	0.91 (0.43–1.92)	0.8017	1.08 (0.46–2.57)	0.8610
6 +	**2.58 (1.01–6.62)**	**0.0481**	1.68 (0.59–4.83)	0.3343	0.99 (0.25–3.91)	0.9928
Mini Nutritional Assessment (ref. normal nutritional status)						
Risk of malnutrition	**3.10 (1.52–6.35)**	**0.0020**	1.45 (0.66–3.20)	0.3561	1.00 (0.35–2.81)	0.9923
Malnutrition	**2.01 (1.26–3.21)**	**0.0036**	1.21 (0.69–2.11)	0.5040	1.29 (0.65–2.55)	0.4619

*Abbreviations: OR* odds ratio, *CI* confidence interval, *ref* reference

Boldface indicates statistical significance

^a^*n* = 391 residents were included in this model

^b^*n* = 390 residents were included in this model

^c^*n* = 391 residents were included in this model

With respect to emergency medical care utilization, residents aged 85 + vs. 60–74 years (2.58 [1.14–5.84]) and those with LTC grades 3 or 4/5 vs. 1/2 (2.65 [1.07–6.55], 6.31 [2.60–15.35]) were more likely to utilize this medical care type. Furthermore, residents who had spent 5 + vs. 1- < 3 years in the respective nursing home were less likely to utilize emergency medical care (0.46 [0.24–0.86]). For sex, number of Elixhauser diseases, and nutritional status, no associations were found.

Regarding contacts with the regional on-call medical services, no associations with individual characteristics were observed.

## Discussion

This research found that nearly half of the residents had been hospitalized, about a quarter had utilized emergency medical care, and around one-tenth had had contact with a regional on-call medical service at least once in the previous 12 months. Men, older residents, those with higher LTC grades, individuals with a higher number of Elixhauser diseases, and those at risk of or with malnutrition were more likely to be hospitalized compared to the respective reference group. Emergency medical care utilization was more frequent among older residents and those with higher LTC grades compared to their counterparts, and less frequent among individuals who had already resided in the respective nursing home for five or more years. Regarding the on-call medical services, no significant associations were found.

### Hospitalizations

Compared to the international literature [29], our proportion of hospitalized residents (46%) is higher than those reported for a time period of one year in nursing home populations with comparable sex and age distributions from the USA (25–35%) [49, 50], Canada (26%) [51], and Italy (12%) [52]. Our proportion is, however, comparable to another German study [28], which found that 43% of residents had been hospitalized at least once in the previous 12 months. Furthermore, our mean number of 1.6 hospitalizations among residents with at least one hospitalization is also comparable with the 2.0 reported for LTC dependents previously based on German nationwide data [13]. Generally, the frequent hospitalizations among nursing home residents in Germany are a well-known phenomenon, particularly at the end of life [23, 53, 54], whereas our mean hospitalization length of six days is internationally more comparable, for example with American [55] and Swedish [56] studies reporting seven days.

Our finding that male residents were more likely to be hospitalized than female residents is in line with previous international and German studies [23, 26, 29, 57–59]. Although the reasons for this association have only been researched to a limited extent [26, 29], it is reported that male residents generally receive more burdensome interventions (transitions of care, invasive procedures, and physical restraints) and potentially burdensome antibiotic therapy at the end of life than female residents [58]. Given these results, future studies should investigate whether sex differences in hospitalizations among nursing home residents can be explained by different medical care needs or whether male residents are more likely to be inappropriately hospitalized than female residents.

With respect to age differences, we found that older residents are more likely to be hospitalized than younger residents. According to a systematic review including 21 studies [29], however, the effect of age on hospitalizations is not entirely clear: While some studies reported a positive association, others reported no or even a negative association, and the latter appears to be relatively clear regarding hospitalizations at the end of life, as reported in another systematic review [26]. Generally, further research on the association between age and hospitalizations among nursing home residents is needed that differentiates between different phases of life, which has rarely been done so far.

Regarding LTC grades, our results point in the same direction as those of existing German studies [12, 13], and with respect to the number of Elixhauser diseases our findings are plausible given that the Elixhauser comorbidity measure was originally developed to predict inpatient outcomes [42]. Furthermore, studies from Sweden [60] and Italy [61] reported that residents with malnutrition have an increased risk of being hospitalized, which is in line with our findings. This is an important aspect because in our study population, almost every second resident was malnourished or at risk of malnutrition, and similar prevalences were observed before [62, 63].

### Emergency medical care utilization

In our research, emergency medical care was utilized by 23% of residents in the previous 12 months, with a mean number of 1.3 emergency contacts per resident among those with at least one contact. When comparing these results to the literature it should be taken into consideration that our definition of emergency medical care includes both emergency medical care utilized in nursing homes and emergency department visits, whereas most existing studies [14, 27, 64–66] focus only on emergency department visits. In these studies, the reported proportions of residents with at least one emergency department visit within one year vary greatly by country. They were lowest in the Netherlands (8%) [65], followed by Germany (21–27%) [14, 65], Sweden (29%) [67], and the USA (47–62%) [68, 69]. Furthermore, the reported mean numbers of emergency department visits are difficult to compare with our analysis because besides the definition of emergency medical care the observation periods [64] and denominators [66] of existing studies also differ from our research.

Our finding that the likelihood of emergency medical care utilization was positively associated with age is comparable with an existing German study focusing on emergency department visits [57], but it should also be borne in mind that three systematic reviews [24, 27, 70] indicate an unclear association between age and emergency department visits. With respect to the positive association with LTC grades and the negative association with years of residence in the respective nursing home, our results are also comparable with a previous German study [57]. Furthermore, two systematic reviews [24, 70] and an additional German study [14] found a higher likelihood of emergency department visits in the first months after moving into a nursing home than thereafter. Regarding sex, we found no significant association, but our results suggest that male residents were more likely to utilize emergency medical care than female residents, which is in line with previous international and national studies [14, 24, 27].

### Contacts with regional on-call medical services

With respect to contacts with the on-call medical services, which have rarely been investigated so far, we found that 12% of residents had had at least one contact in the previous 12 months, with a mean number of 1.2 contacts among those with contact. A previous German study [14] reported a proportion of 28% with contact, but focused only on the first year after moving into a nursing home, when the proportion can be expected to be higher than thereafter, as is the case with emergency medical care utilization [14, 24, 70]. In this regard it should also be noted that in our research, though not statistically significant, the likelihood of contact with the on-call medical services decreased with increasing years of residence in the respective nursing home. Furthermore, another German study [57] observed a proportion of 33% (545/1665) of residents who had contact with an on-call medical service within two years, which is slightly more comparable with our proportion for only one year. Although in our research the likelihood of contacts with the on-call medical services was not associated with individual characteristics of residents, other German studies found that male residents were more likely to have contact with the on-call medical services than female residents [14, 57].

Overall, the reason for the rare contacts between the on-call medical services and nursing home residents may be an insufficient availability of doctors on call who are able to visit nursing homes immediately, so that emergency medical care utilization or even hospitalizations become necessary. Given the attempts to provide more acute medical care directly in nursing homes [71–74], future studies should investigate whether strengthening medical care provision by on-call doctors and other professionals can contribute to a reduction of hospitalizations and emergency medical care utilization among nursing home residents.

### Strengths and limitations

A major strength of this research is that more than 400 residents from 44 nursing homes were included, covering the heterogeneity in the utilization of acute medical care. A further strength is that proportions of residents with utilization of three different acute medical care types as well as respective utilization numbers were systematically investigated and compared among the same group of residents. Moreover, acute medical care utilization in the previous 12 months was examined, allowing potential seasonal variations in utilization patterns over the course of the year. Another strength is the standardized data collection, which was performed by trained study nurses and included a review of nursing records as well as the established MNA-SF.

However, some limitations should also be noted. First, only residents who consented to participate were included, which introduces the possibility of selection bias. In this regard it is important to note that a non-response analysis [38] showed that residents who participated in the cross-sectional data collection of MVP-STAT were younger, less likely to have contact with general practitioners, and less likely to die in the year of data collection than nonparticipants, whereas regarding sex, LTC grades, number of hospitalizations, and selected diagnoses no differences were found. Second, only individuals who had resided in the respective nursing home for at least 12 months were included. Because a previous German study [75] reported that the proportion of residents with at least one hospitalization is higher in the first six months after moving into a nursing home than thereafter, our results may not be extrapolatable to residents who died shortly after nursing home admission. However, our results on acute medical care utilization in the previous 12 months cannot be biased by different utilization patterns immediately before nursing home admission. It is important to point this out because the previous German study [75] also showed that the proportion of residents who were hospitalized is particularly high in the six months before nursing home admission. Furthermore, the duration of residence in our study is only slightly more than one year higher than in another German study [33] including all residents of the participating care units of 21 nursing homes (mean 4.4 years [SD 4.3] vs. 3.2 years [3.4]). Third, the collected data included no differentiation between unplanned and planned hospitalizations, nor any differentiation between emergency medical care utilization in nursing homes and emergency departments, which complicates the interpretation of our results. However, differentiating between planned and unplanned hospitalizations among nursing home residents is often difficult, and most existing studies have not made this differentiation either. Fourth, the reviewed nursing records might not always have been complete in relation to acute medical care utilization and diagnoses. For example, we did not consider dementia in our analyses because the documented dementia prevalence was only 35%, whereas analyses of German claims data reported a prevalence between 52% [76] and 60% [77] among nursing home residents. Fifth, because the utilization of acute medical care was not analyzed in relation to individual needs, we were unable to investigate whether the utilization was appropriate. Sixth, our research is based on data from residents in nursing homes in Bremen and Lower Saxony, resulting in limited geographical coverage that might limit the generalizability of our results to other regions. However, our distributions of ownership, sex, age, and LTC grades are comparable with nationwide German data (51% vs. 54% independent nonprofit nursing homes, 44% vs. 41% private nursing homes, 5% vs. 5% public/municipal nursing homes, 67% vs. 70% female residents, 48% vs. 50% 85 + years old residents, and 43% vs. 45% residents with LTC grades 4/5) [78]. Furthermore, our mean number of nursing home beds per participating facility is only slightly higher than in nationwide data (87 vs. approximately 77 beds) [78]. Finally, the analyzed data were collected in 2018/2019 and relate to the 12 months prior to data collection. Therefore, we were unable to investigate the impact of the implementation of Advance Care Planning in German nursing homes (§ 132 g Social Code Book V), which started in 2018, on our results [79, 80]. However, ongoing research is addressing this important research gap [81].

## Conclusions

Almost half of the nursing home residents had been hospitalized, approximately a quarter utilized emergency medical care, and around a tenth had contact with a regional on-call medical service at least once in the previous 12 months. The frequent hospitalizations and emergency medical utilization along with comparatively few contacts with the on-call medical services underscore the potential to improve medical care among nursing home residents. Future studies should investigate why residents in Germany are hospitalized more frequently than those in many other countries, and whether hospitalizations and emergency medical care utilization among residents can be reduced by strengthening medical care provision by on-call doctors and other professionals.

## Data Availability

The datasets generated and/or analyzed during the current study are not publicly available due to data protection, but data sharing is feasible upon reasonable request and in collaboration with the authors and Competence Center for Clinical Trials of the University of Bremen.

## Abbreviations

CI: Confidence interval
ICD-10: International classification of diseases, 10th revision
LTC: Long-term care
MNA-SF: Mini nutritional assessment short-form
MVP-STAT: Needs-based provision of medical care to nursing home residents
OR: Odds ratio
SD: Standard deviation
